# Herbal compound 861 prevents hepatic fibrosis by inhibiting the TGF-β1/Smad/SnoN pathway in bile duct-ligated rats

**DOI:** 10.1186/s12906-018-2119-7

**Published:** 2018-02-05

**Authors:** Cheng Chi, Xiao-ya Liu, Fei Hou, Xiao-zheng Yu, Chun-yun Li, Li-jian Cui, Rui-xia Liu, Cheng-hong Yin

**Affiliations:** 1 Department of Internal Medicine, Beijing Obstetrics and Gynecology Hospital, Capital Medical University, Beijing, 100026 China; 2 Department of Emergency, Beijing Chao-Yang Hospital (Jingxi Campus), Capital Medical University, Beijing, 100043 China; 3 Department of Central Laboratory, Beijing Obstetrics and Gynecology Hospital, Beijing, 100026 China

**Keywords:** Herbal compound 861, Hepatic fibrosis, Ski-related novel protein N, TGF-β1

## Abstract

**Background:**

This study was to evaluate the effects of herbal compound 861 (Cpd861) on ski-related novel protein N (SnoN) and transforming growth factor-β1 (TGF-β1) /Smad signaling in rats with bile duct ligation (BDL)-induced hepatic fibrosis, and to explore the mechanisms of Cpd861 on hepatic fibrosis.

**Methods:**

Thirty Wistar male rats were randomly divided into three groups: sham operation, BDL, and Cpd861. To induce hepatic fibrosis, BDL and Cpd861 group rats underwent bile duct ligation. Cpd861 at 9 g/kg/d or an equal volume of normal saline was administered intragastrically for 28 days. Liver injury was assessed biochemically and histologically. Protein and mRNA changes for SnoN and TGF-β1/Smad signaling (TGF-β1, Smad2, phosphorylated Smad2 [p-Smad2], phosphorylated Smad3 [p-Smad3], fibronectin, and collagen III) were determined by Western blotting and quantitative real-time PCR.

**Results:**

BDL rats treated with Cpd861 had significantly alleviated hepatic fibrosis compared to BDL rats not receiving Cpd861 treatment. Moreover, Cpd861 decreased the expression of fibrosis-associated proteins fibronectin and collagen III in liver tissue. Cpd861 administration increased the expression of SnoN protein, did not change SnoN mRNA level, and decreased TGF-β1, p-Smad2, and p-Smad3 protein expression compared to BDL without Cpd861 treatment.

**Conclusions:**

Cpd861 attenuates hepatic fibrosis by increasing SnoN protein expression and inhibiting the TGF-β1/Smad signaling pathway.

## Background

Hepatic fibrosis, which results from dysregulation of normal healing and scar formation, occurs commonly among patients with hepatic cirrhosis or dysfunction [1]. The histologic characteristic of hepatic fibrosis is increased extracellular matrix (ECM) components, primarily collagen. Fibrous scarring and liver parenchymal cell destruction also occur [2, 3]. It is thought that hepatic fibrosis is reversible [4], but the resulting structural damage to liver lobules and vasculature leads to cirrhosis, which is irreversible. Therefore, prevention and reversal of hepatic fibrosis is necessary to treat various chronic liver diseases and prevent cirrhosis.

Many studies have confirmed that transforming growth factor β1 (TGF-β1) is the most effective and powerful cytokine promoter of hepatic fibrosis [5, 6]. As the primary cytokine regulating hepatic satellite cell (HSC) function, TGF-β1 activates, promotes, and stimulates HSC proliferation and differentiation by phosphorylating downstream signaling molecules Sma- and Mad-related proteins 2 and 3 (Smad2 and Smad3). HSCs are the main cells produced ECM. Therefore, the TGF-β1/Smad signaling pathway plays an important role in hepatic fibrosis. Recent studies show that Ski-related novel protein N (SnoN) can block TGF-β1/Smad signaling in cell cytoplasm and nuclei, thereby regulating the TGF-β1 signaling pathway tightly [7–9].

Multiple treatment strategies are aimed at alleviating hepatic inflammatory necrosis and minimizing the severity of hepatic fibrosis. Among these strategies, herbal compound 861 (Cpd861) is a patented formulation derived from traditional recipes and developed by Professor Wang Baoen. The anti-fibrotic activity of Cpd861 was demonstrated by in vivo and vitro experiments of fibrinogenesis and related proteins, including α-smooth muscle actin (α-SMA), collagen I, and collagen III [10–12]. Our most recent study [13] focused on bone morphogenetic protein 7 (BMP-7)/Smad signaling to determine its biochemical mechanisms involved in attenuating liver fibrosis. BMP-7 is a member of the strongly antifibrotic BMP family, whose members have shown antagonism against TGF-β-dependent fibrogenesis. Other studies support the role of Cpd861 inhibit TGF-β1 expression, HSC proliferation and activation, and hepatic fibrosis progression [13]. However, the antifibrotic molecular mechanism of Cpd861 remains unknown. Bile duct ligation (BDL) surgery is widely used to simulate human hepatic fibrosis in rats. The present study was designed to further investigate whether Cpd861 affects the expression of TGF-β1/Smad signaling pathway and its negative regulation factor SnoN in a rat hepatic fibrosis model.

## Methods

### Drugs

Cpd861 is comprised of *Salvia miltiorrhiza, astragalus, Radix bupleur, Caulis spatholobi, Szechwan lovage rhizome, Rhizoma cyperi, Radix Paeoniae Rubra, tangerine peel, angelica,* and *safflower.* The medical preparation is used clinically at the Beijing Friendship Hospital of Capital Medical University and is manufactured by Beijing Yadong Pharmaceutical Co., Ltd. in 18 g packets (Beijing health and medicine manufacturing and processing number Z20063100).

### Establishment of animal model and drug administration

Animal care and experimental procedures were approved by the Ethics Committee of Beijing Friendship Hospital, Capital Medical University (Beijing, China). Male Wistar rats in specific pathogen free (SPF) grade weighing 180 ± 20 g were purchased from the Institute of Laboratory Animal Science, Chinese Academy of Medical Sciences, and Peking Union Medical College (Beijing, China; certificate number Jing 2009–0007).

The rats were housed under specific pathogen-free conditions with controlled lighting (12 h per day) and temperature (22 ± 2 °C) and fed standard laboratory chow and allowed water ad libitum at the animal laboratories of Beijing Friendship Hospital. After 1 week of adaptive feeding, food was withheld for 12 h prior to modeling. Rats were anesthetized with pentobarbital sodium (40 mg/kg; Sigma-Aldrich, St. Louis, MO, USA). The common bile duct was then exposed and ligated to induce fibrosis by total biliary obstruction according to a previously described method [14, 15]. After BDL surgery, the rats were maintained for 4 weeks (*n* = 10 per group) with free access to food and water. A second group of rats that were treated similarly except they underwent a sham operation without BDL were used as controls. Laparotomy was performed in the sham operation group, and the common bile duct was exposed without ligation.

Beginning 1 week after BDL, 10 rats in the Cpd861 group received Cpd861 by gavage once daily for 4 weeks at a dose of 9 g/kg/d (equivalent of 10 times the recommended dose for human adults). The BDL and sham operation groups received normal saline at a daily dose of 0.25 ml/ 100 g by gavage. At 4 weeks, rats were anesthetized by intraperitoneal injection of sodium pentobarbital (40 mg/kg). Blood samples were collected from the heart. The rats were sacrificed by dislocation of the cervical spine. Then the right lobe of the liver was collected. Liver and serum samples were kept frozen at − 80 °C until being assayed.

### Histologic examination

Liver samples were fixed in 4% paraformaldehyde, paraffin embedded, and sectioned at 4 μm. Histologic changes were assessed using hematoxylin-eosin (H&E) and Masson’s trichrome staining (Bestbio Co., Shanghai, China). Each sample was independently assessed by a pathologist who was blinded to the study protocol. Samples were scored according to the METAVIR scoring system published by Bedossa and Poynard [16]: (1) F0 = no fibrosis; (2) F1 = portal fibrosis without septa; (3) F2 = portal fibrosis with few septa; or (4) F4 = cirrhosis. Histologic images were obtained for each group using a Leica DM6000B microscope (Leica Microsystems GmbH, Wetzlar, Germany).

### Liver function test

Blood samples were centrifuged at 4000×*g* for 5 min to separate the serum from other blood components. The samples were subsequently used to determine aspartate aminotransferase (AST), alanine aminotransferase (ALT), total bilirubin (TBIL), and direct bilirubin (DBIL) levels using an automatic biochemistry analyzer (Abbott Laboratories, Chicago, IL, USA), according to the manufacturer’s protocol.

### Analysis of TGF-β1, Smad2, Smad3 and SnoN mRNA expression by quantitative real-time polymerase chain reaction

Total RNA was extracted from livers using TRIzol (Invitrogen, Waltham, MA, USA) and reverse transcribed with a reverse transcriptional kit (Fermentas, Burlington, ON, Canada). The resultant cDNA was subjected to quantitative real-time polymerase chain reaction (qRT-PCR), and primer sequences for TGF-β1, Smad2, Smad3, SnoN, and housekeeping gene glyceraldehyde-3-phosphate dehydrogenase (GAPDH) were designed (Table 1). qRT-PCR was performed using the Power SYBR Green PCR Master Mix using a 7500 real-time PCR instrument (reagent and equipment manufactured by Applied Biosystems, Foster City, CA, USA). The PCR protocol was as follows: denaturation at 95 °C for 5 min, then 95 °C for 15 s, and 60 °C for 1 min for 40 cycles. The amount of GAPDH cDNA was used to normalize the sample amounts used for determinations.

**Table 1:** PCR primer sequences for TGF-β1, Smad2, Smad3, SnoN and GAPDH

Gene	Forward sequence	Reverse sequence
TGF-β1	5’-ATACGCCTGAGTGGCTGTCT-3’	5’-TGGGACTGATCCCATTGATT-3’
Smad2	5’-TGAGCTTGAGAAAGCCATCA-3’	5’-TGTGTCCCACTGATCTACCG-3’
Smad3	5’-CATTACCATCCCCAGGTCAC-3’	5’-CGTAACTCATGGTGGCTGTG-3’
SnoN	5’-CCATTCAATGCCCCATCCT-3’	5’-AGTTCGTGGCCGCAATAAAG-3’
GAPDH	5’-CCTGCCAAGTATGATGACATCAAGA-3’	5’-GTAGCCCAGGATGCCCTTTAGT-3’

GADPH, glyceraldehyde-3-phosphate dehydrogenase; Smad2, Sma- and Mad-related protein 2; Smad3, Sma- and Mad-related protein 3; SnoN, ski-related novel protein N; TGF-β1, transforming growth factor-β1

### Analysis of TGF-β1 and SnoN protein expression by immunohistochemistry

Histologic sections were heated for 60 min at 60 °C, deparaffinized using xylene, and rinsed in a decreasing alcohol gradient (100%, 95%, and 70%). Subsequently, antigens were retrieved with citrate buffer (0.01 M; pH 6) in a microwave oven. Following treatment with hydrogen peroxide at 37 °C for 30 min to block endogenous peroxidase activity, sections were incubated with a primary antibody overnight at 4 °C and subsequently incubated with a peroxidase conjugated monoclonal goat anti-rabbit immunoglobulin G secondary antibody (PV-9002; Zhongshan Goldenbridge Biotechnology Co., Ltd., Beijing, China) for 30 min at room temperature. Finally, sections were stained with diaminobenzidine (Zhongshan Goldenbridge Biotechnology Co., Ltd.) for 1 min then counterstained with hematoxylin for 20 s. Polyclonal rabbit anti-rat SnoN (1:200 dilution; cat. no. ab78979; Abcam, Cambridge, UK) and polyclonal rabbit anti-rat TGF-β1 (1:50 dilution; sc-398; Santa Cruz Biotechnology, Dallas, TX, USA) were used as primary antibodies.

### Analysis of TGF-β1, p-Smad2, p-Smad3, SnoN, collagen III and fibronectin protein expression by western blotting

The total protein was extracted by homogenizing 100 mg of liver tissue using RIPA Lysis (Thermo Fisher Scientific, Waltham, MA, USA) supplemented with phenylmethylsulfonyl fluoride. Tissue lysates were centrifuged at 1811×*g* for 10 min at 4 °C. Immediately thereafter, the supernatants were harvested and stored at − 80 °C until further analysis. Protein concentrations were determined using a BCA Protein Assay Kit (Applygen Technologies Inc., Beijing, China). Total proteins were separated using 10% sodium dodecyl sulfate-polyacrylamide gels and then transferred to polyvinylidene fluoride membranes (Merck Millipore, Billerica, MA, USA). After blocking with 5% skimmed milk for 2 h at room temperature, membranes were incubated with rabbit anti-mouse TGF-β1 (1:200; Abcam), p-Smad2 (1:500; Santa Cruz Biotechnology), p-Smad3 (1:500; Santa Cruz Biotechnology), SnoN (1:400; Santa Cruz Biotechnology), collagen III (1:100; Abcam) and fibronectin (FN; 1:500; Santa Cruz Biotechnology) overnight at 4 °C. β-actin was used as a loading control with a mouse monoclonal antibody (1:400; Abcam). Peroxidase-conjugated anti-rabbit or anti-mouse secondary antibodies (1:10,000; Santa Cruz Biotechnology) were also used. Bound antibodies were visualized using enhanced chemiluminescence Western blot detection reagents (Merck Millipore) were analyzed with Image Lab software (Bio-Rad Laboratories, Hercules, CA, USA).

### Statistical methods

Statistical analyses were performed using SPSS software version 17.0 (IBM, New York, NY, USA). Normally distributed data were described as means ± standard deviations. One-way analysis of variance (ANOVA) was used to compare independent samples of multiple groups. For all tests, a *P* value less than 0.05 was considered statistically significant.

## Results

### Morphologic observations of gross specimens

**Fig. 1:**
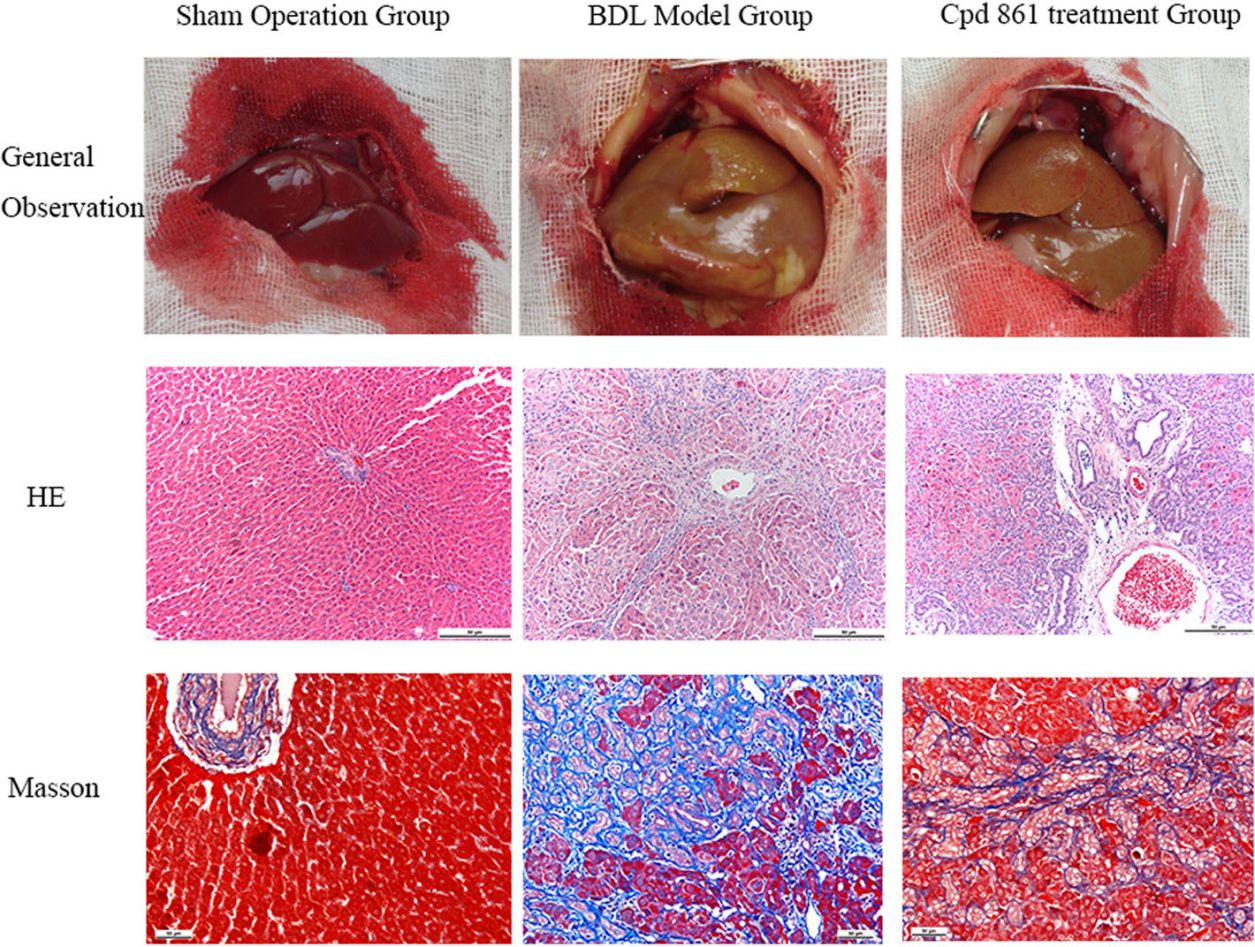
Gross specimen morphology for sham operation, bile duct ligation, and Cpd861 groups. Histologic examination of liver sections was performed using H&E and Masson’s trichrome staining (magnification, × 200)

Rats were sacrificed after 28 days’ treatment with Cpd861. Livers from control rats were soft, ruddy, shiny, smooth, and delicate, with sharp edges (Fig. 1). In contrast, livers from model rats were severely damaged, with yellowing, adhered coverings, uneven surfaces, blunt edges, and shrunken size. The Cpd861 treatment group had livers that were yellowed with fewer surface tubercles; however, the yellowing was less pronounced compared to the model group, and there was no liver shrinkage.

### Cpd861 effects on collagen deposition and tissue damage in rats undergoing BDL

**Fig. 2:**
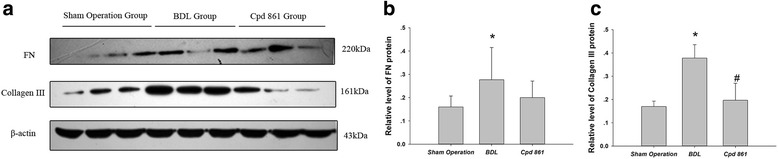
**a** Collagen III, fibronectin, and β-actin expression levels, as determined by Western blot, with β-actin used as a loading control. Quantification of Western blot results for relative levels of (**b**) fibronectin and (**c**) collagen III. Data are expressed as means ± standard deviations (*n* = 6). One-way analysis of variance was used for statistical comparisons. * *P* < 0.05 vs sham operation group; ^#^ *P* < 0.05 vs BDL group

In the sham operation group, there was normal lobular architecture with central veins, radiating hepatic cords, and no regenerated collagen fibers (Fig. 1). In contrast, livers from the BDL group showed collagen fibers, fibroblasts, and small capillaries forming membrane-like intervals, with adjacent cellular hyperplasia and resultant pseudo-lobule formation. Compared to BDL livers, those from the Cpd861 group had decreased collagenous fiber and significantly ameliorated pathology. Using the METAVIR system, the mean fibrosis score was significantly lower in the Cpd861 group compared to the BDL and sham operation groups (Table 2). As with the H&E and Masson’s staining, Western blot also showed that BDL induced cholestatic hepatic fibrosis by increasing FN (0.16 ± 0.05 vs 0.28 ± 0.14, *P* < 0.05) and collagen III (0.17 ± 0.02 vs 0.38 ± 0.06, *P* < 0.05) expression (Fig. 2a-c). Furthermore, Cpd861 was associated with decreased protein collagen III expression (0.38 ± 0.06 vs 0.19 ± 0.07, *P* < 0.05; Fig. 2a and b).

**Table 2:** Liver fibrosis stage using the METAVIR system

Group	n	liver fibrosis stage				
		F0	F1	F2	F3	F4
Sham operation group	6	6	0	0	0	0
BDL model group*	6	0	0	1	3	2
Cpd 861 group*^#^	6	0	2	2	2	0

Values are presented as stage scores (*n* = 6). Chi-square tests were used for statistical comparisons. *P < 0.05 vs sham operation group; ^#^*P* < 0.05 vs bile duct ligation group

### Serum biochemical parameters in bile duct ligation model rats

Compared to rats in the sham operation group, those in the BDL group had increased serum levels of ALT, AST, TBIL, and DBIL (all *P* < 0.05; Table 3). Although rats in the Cpd861 group did not differ with respect to TBIL and DBIL levels, Cpd861 did have reduced ALT and AST levels compared to BDL rats.

**Table 3:** Effect of Cpd 861 on serum biochemical indicators

Group	TBIL(μmol/L)	DBIL(μmol/L)	ALT(U/L)	AST(U/L)
Sham operation group	0.87 ± 0.21	0.51 ± 0.38	34.88 ± 3.00	115.38 ± 16.07
BDL group	88.14 ± 35.86*	79.74 ± 32.63*	93.75 ± 34.30*	360.50 ± 175.07*
Cpd 861 group	118.81 ± 22.93*	99.66 ± 7.73*	86.00 ± 22.86*	320.25 ± 140.62*

Values are presented as means ± standard deviations (*n* = 6). One-way analysis of variance was used for statistical comparisons. *P < 0.05 vs sham operation group

### Effect of Cpd 861 on TGF-β1/Smad signaling-associated genes in BDL model rats

**Fig. 3:**
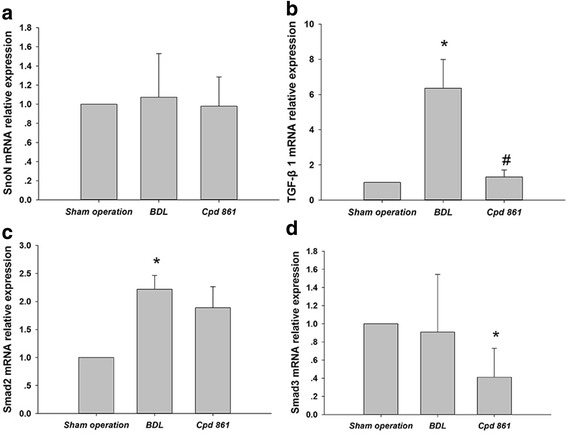
Effect of Cpd861 on gene expression of SnoN, TGF-β1, Smad2, and Smad3 for sham operation, bile duct ligation, and Cpd861 groups, as detected by reverse transcription quantitative polymerase chain reaction. Relative gene expression levels for (**a**) SnoN, (**b**) TGF-β1, (**c**) Smad2, and (**d**) Smad3. Data are expressed as means ± standard deviations (*n* = 6). One-way analysis of variance was used for statistical comparisons. * *P* < 0.05 vs sham operation group; ^#^ *P* < 0.05 vs BDL group

To assess the anti-fibrotic mechanism of Cpd861, the effect of Cpd861 on the expression of TGF-β1/Smad signaling-associated genes was first assessed. The Cpd861 group had decreased TGF-β1 gene expression compared to the BDL group (*P* < 0.05) but not the sham operation group (*P* > 0.05; Fig. 3b). Smad2 gene expression did not differ among the three groups (*P* > 0.05, Fig. 3c). Cpd861 treatment was associated with decreased gene expression of Smad3 mRNA compared to the sham operation (*P* < 0.05, Fig. 3d). There was no difference in SnoN gene expression among the three groups (*P* > 0.05, Fig. 3a).

### Histologic assessment of liver tissue

**Fig. 4:**
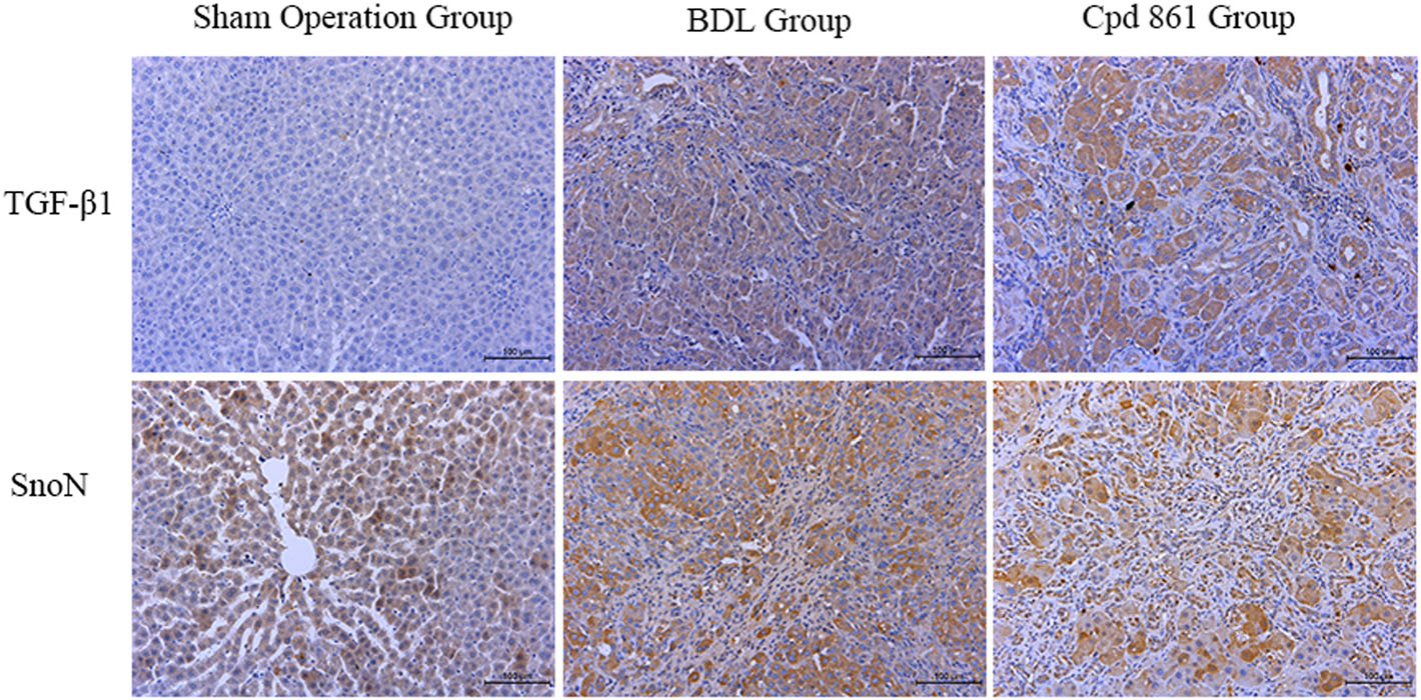
Positive expression location of TGF-β1 and SnoN for sham operation, BDL, and Cpd861 groups, as analyzed by immunohistochemistry assay (magnification, × 200)

Positive immunostaining for TGF-β1was observed in hepatocyte nuclei, but was especially pronounced in hepatocyte cytoplasm (Fig. 4). Furthermore, SnoN expression, which was observed in all three groups, occurred predominantly in the cytoplasm (Fig. 4).

### Effect of Cpd 861 on TGF-β1/Smad signaling-associated proteins in bile duct ligation model rats

**Fig. 5:**
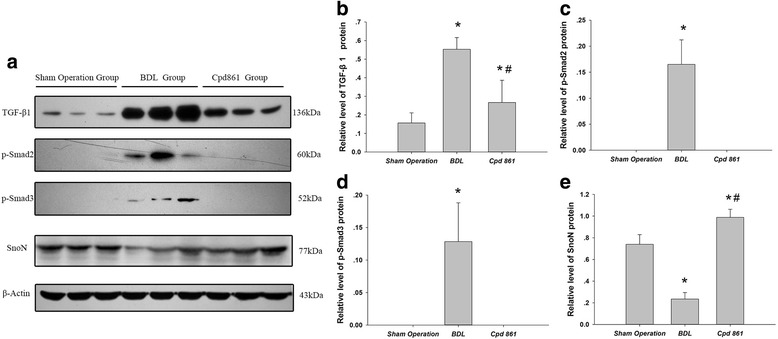
**a** Expression levels of TGF-β1, p-Smad2, p-Smad3, SnoN, and β-actin were detected by Western blot, with β-actin used as a loading control. Quantification of Western blots for relative levels of (**b**) TGF-β1, (**c**) p-Smad2, (**d**) p-Smad3, and (**e**) SnoN. Data are expressed as means ± standard deviations (*n* = 6). One-way analysis of variance was used for statistical comparisons. ^*^ *P* < 0.05 vs sham operation group; ^#^ *P* < 0.05 vs BDL group

Semi-quantitative analysis of Western blots revealed increased expression of TGF-β1 (0.55 ± 0.06 vs 0.16 ± 0.05, *P* < 0.05), p-Smad2 (0.16 ± 0.04 vs 0.00 ± 0.00, *P* < 0.05), and p-Smad3 (0.13 ± 0.06 vs 0.00 ± 0.00, *P* < 0.05; Fig. 5a-d). However, SnoN expression was decreased in the BDL group compared to the sham operation group (0.24 ± 0.59 vs 0.74 ± 0.89, *P* < 0.05; Fig. 5e). Compared with BDL model rats, those receiving Cpd861 had decreased expression of TGF-β1 (0.27 ± 0.12 vs 0.55 ± 0.06, *P* < 0.05, Fig. 5b), p-Smad2 (0.00 ± 0.00 vs 0.17 ± 0.04, *P* < 0.05), and p-Smad3 (0.00 ± 0.00 vs 0.13 ± 0.06, *P* < 0.05; Fig. 5c and d) and increased expression of SnoN (0.99 ± 0.07 vs 0.24 ± 0.06, *P* < 0.05; Fig. 5e).

## Discussion

Hepatic fibrosis is a complex pathophysiologic process associated with various growth factors and signaling pathways. Among them, the TGF-β1/Smad signaling pathway is considered integral to fibrogenesis because it activates HSCs [17]. Recent studies show that SnoN may regulate the TGF-β1 signaling pathway tightly [18, 19].

In our study, Cpd861 efficacy was examined in a BDL-induced hepatic fibrosis rat model. H&E and Masson’s staining results suggest that pathologic liver cell damage was most pronounced for the BDL group and that Cpd861 could significantly reduce pathologic changes to liver lobules and pseudolobules in fibrotic rat livers. Moreover, our finding of decreased serum ALT and AST in the Cpd861 group compared to the BDL group suggests that liver injury can be attenuated by therapeutic intervention. Although the mean values for total bilirubin and direct bilirubin were larger in the Cpd861 group compared to the BDL group, the difference was not statistically significant. Our previous work demonstrated the effect of Cpd861 on tissue inhibition of metalloproteinase1 gene expression in HSC-T6 cells [20] and quantified matrix metalloprotein-2 mRNA levels and activity in rat model liver fibrosis [21]. We also showed that Cpd861 could attenuate liver fibrosis through upregulation of BMP-7/Smad signaling [13]. Taken together with the current findings, there is good evidence that Cpd861 protects liver function and ameliorates hepatic fibrosis.

It is widely acknowledged that TGF-β1 is the most potent cytokine fostering hepatic fibrosis; through Smad2 and Smad3 phosphorylation, it activates HSCs to produce collagen III and FN. Once associated with its receptor, TGF-β1 activates receptor-mediated Smads via phosphorylation of “SSXS” motifs at COOH-termini. This process releases receptor-mediated Smads (R-Smads) from the Smad anchor, which allows their complex formation with comediator Smad4 and direct binding to nuclear DNA to alter fibrosis-related gene expression. In our study, Cpd861 administration led to decreased TGF-β1, p-Smad2, and p-Smad3 protein levels and decreased TGF-β1 mRNA expression. These results suggest that TGF-β1 gene and protein downregulation from Cpd861 treatment is followed by decreased p-Smad2 and p-Smad3 expression. TGF-β1 is a key activator of HSC proliferation, which may be associated with hepatic fibrosis [22].

SnoN, a member of the Ski-Sno family, negatively regulates the TGF-β1 signaling pathway [8]. Recent studies have demonstrated that SnoN could block cytoplasmic and nuclear TGF-β1/Smad signaling and tightly regulate TGF-β1 activity [9]. SnoN inhibits TGF-β1/Smad signaling pathway in three ways. Firstly, nuclear SnoN disrupts the interaction of phosphorylated R-Smad2/3 with comediator Smad4 by forming a wedge between the complexes [23]. Secondly, the SnoN-Smad complex recruits transcriptional co-repressors, including nuclear receptor co-repressors homeodomain interacting protein kinase2, mSin3A, and histone deacetylase, to repress gene transcription [24]. Finally, SnoN prevents coactivator binding of p300 and CREB-binding protein to the Smad heterocomplex [25]. Recent studies show that blocking the TGF-β1 receptor could increase SnoN expression [26–28]. In our study, SnoN expression in Cpd861-treated rats increased significantly, indicating that SnoN served as an alternate target to regulate TGF-β1 signaling when the usual pathway was interrupted by Cpd861. Interestingly, our study also demonstrated no effect of Cpd861 on SnoN mRNA expression. The phenomenon by which SnoN protein is increased without change to its mRNA is believed to be related to decreased ubiquitination and degradation of SnoN protein. Further study is needed to explain why SnoN expression is increased in BDL rats who receive Cpd861. Other mechanisms, especially those related to hepatocyte protective effects of Cpd861 in hepatic fibrosis, require further research.

## Conclusions

Our study demonstrated that Cpd861 can attenuate hepatic fibrosis and its effects on liver function by decreasing Smad2 and Smad3 protein levels while increasing SnoN protein levels. The effect of Cpd861 on p-Smad2, p-Smad3, and SnoN expression show that these proteins may be treatment targets for hepatic fibrosis. Our study provides further support for Cpd861 as an attenuator of hepatic fibrosis through downregulation of the TGF-β1/Smad/SnoN signaling pathway.

## Abbreviations

α-SMA: α-smooth muscle actin
ALT: Alanine aminotransferase
AST: Aspartate aminotransferase
BDL: Bile duct ligation
BMP-7: Bone morphogenetic protein 7
Cpd861: Herbal compound 861
DBIL: Direct bilirubin
ECM: Extracellular matrix
FN: Fibronectin
GAPDH: Glyceraldehyde-3-phosphate dehydrogenase
H&E: Hematoxylin-eosin
HSC: Hepatic satellite cell
qRT-PCR: Quantitative real-time polymerase chain reaction
RIPA: Radio immune precipitation assay
Smad2: Sma- and Mad-related protein 2
Smad3: Sma- and Mad-related protein 3
SnoN: Ski-related novel protein N
TBIL: Total bilirubin
TGF-β1: Transforming growth factor-β1
